# On the Prediction of Upwelling Events at the Colombian Caribbean Coasts from Modis-SST Imagery

**DOI:** 10.3390/s19132861

**Published:** 2019-06-27

**Authors:** José J. Alonso del Rosario, Juan M. Vidal Pérez, Elizabeth Blázquez Gómez

**Affiliations:** 1 Department of Applied Physics, University of Cádiz, CASEM, Avd. Rep. Saharaui s/n, 11510 Puerto Real, Cádiz, Spain; 2 Department of Ship-Building, University of Cádiz, CASEM, Avd. Rep. Saharaui s/n, 11510 Puerto Real, Cádiz, Spain; 3 Department of Earth Sciences, University of Cádiz, CASEM, Avd. Rep. Saharaui s/n, 11510 Puerto Real, Cádiz, Spain

**Keywords:** MODIS-SST, upwelling, Colombian Caribbean, prediction model

## Abstract

The upwelling cores on the Caribbean Colombian coasts are mainly located at the Peninsula de la Guajira and Cabo de la Aguja. We used monthly averaged Moderate Resolution Imaging Spectroradiometer (MODIS) sea surface temperature as the only information to build up a prediction model for the upwelling events. This comprised two steps: (i) the reduction of the complexity by means of the Karhunen–Loève transform and (ii) a prediction model of time series. Two prediction models were considered: (a) a parametric autoregressive-moving average (ARMA) time series from the Box–Jenkins methodology and (b) a harmonic synthesis model. The harmonic synthesis also comprised of two steps: the maximum entropy spectral analysis and a least-squares harmonic analysis on the set of frequencies. The parametric ARMA time series model failed at the time of prediction with a very narrow range, and it was quite difficult to apply. The harmonic synthesis allowed prediction with a horizon of six months with a correlation of about 0.80. The results can be summarized using the time series of the weights of the different oscillation modes, their spatial structures with the nodal lines, and a high confidence model with a horizon of prediction of about four months.

## 1. Introduction

**Figure 1:**
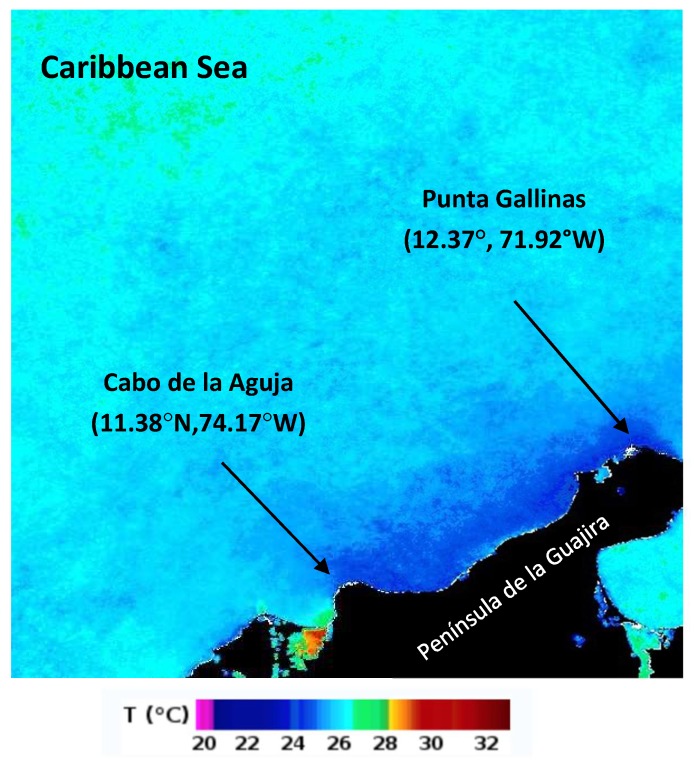
Area under study. The upwelling events are located in the Colombian Caribbean Coast between Punta Gallinas and Cabo de la Aguja at the Colombian Caribbean. The range of temperatures spans from about 20 °C to about 32 °C. The monthly composite image corresponds to December 2003.

One of the nine upwelling areas in the Southern Caribbean [1] is located in Punta Gallinas and Cabo de la Aguja, on the Peninsula de la Guajira (Figure 1). Deep currents travel from the northward branch of the Brazilian current, reaching the Caribbean Sea as the Caribbean current after crossing the Lesser Antilles [2,3]. A description of the upwelling foci and an exhaustive study on the oceanography, nutrient and pigment distribution, and a discussion of the role of the upwelling filaments in the enrichment of open-sea areas in the Caribbean can be found in [4]. Remote sensing studies in the area started with the use of coastal zone color scanner (CZCS) imagery, which showed a low phytoplankton concentration in the open ocean and high concentrations at the coastal areas where upwelling occurs [5]. A study of the upwelling foci along the Southern Caribbean coast using advanced very high resolution radiometer (AVHRR) imagery for the year 1996 can be found in [6]. Upwelling events from AVHRR imagery were also studied in [7], where remote sensing information was combined with meteorological data to link the wind forcing with the sea surface temperature (SST) field. Upwelling events occur twice per year. The most important event occurs when the intertropical convergence zone (ITCZ) is at its southernmost position (December until April–May) with the prevailing trade winds acting in the Southern Caribbean. The second and weaker event occurs in about June–August when the ITCZ is at its northernmost position. This event is caused by intensification of the Caribbean low-level jet (CLLJ) [8,9,10,11].

On the other hand, we studied the upwelling at the Peninsula de la Guajira from several points of view. The application of mathematical morphology [12,13] revealed many internal features of the SST signature. Computation of the fractal dimension of the skeletons of the SST signatures and joint analysis with climate indexes provided insights on the influence of meteorological forcing on the upwelling system. The influence of the upwelling at the Peninsula de la Guajira on some coastal water bodies was studied from a numerical point of view [14].

In this study, we consider the prediction of the upwelling events at the Peninsula de la Guajira from monthly averaged MODIS-SST imagery. Higher frequency composites show cloud cover, and the main objective of this work is the development of a prediction model. This comprises two steps. The first is the reduction of the complexity by means of the Karhunen–Loève transform (KLT). This allows the decomposition of a time series of digital images onto a double-linked basis set consisting of orthonormal spatial modes and their corresponding temporal modes. The second step is the prediction of the temporal modes. This is carried out by two different methods. The Box–Jenkins methodology for time series models (ARMA) produces low-quality predictions. However, the study of frequency composition to apply harmonic synthesis presents some difficulties because of the short length of the time series. The power spectral density (PSD) is computed by maximum entropy spectral analysis (MESA) to avoid spectral leakage of the Fourier transform [15]. The set of frequencies is used to build up a harmonic regression, leading to a high-confidence prediction model.

This manuscript is structured as follows: the descriptions of the available imagery, its pre-processing, a brief introduction of the KLT, some guidelines about reading the information from it, and a description of the time-series prediction model are presented in Section 2. The results and the discussion are presented in Section 3. Finally, the conclusions are drawn in Section 4.

## 2. Materials and Methods

### 2.1. Satellite SST Imagery

The available satellite imagery consisted of a set of 118 monthly composite MODIS-SST images with a 1 km^2^ spatial resolution (December 2002 to September 2012). They were downloaded from [16], but they were originally generated by the Institute of Marine Remote Sensing at the College of Marine Science at University of South Florida. The digital images were cut to the study area (NW corner at [16°N, 77°W], SE corner at [10.5°N, 71.5°W]) to focus the analysis on the Peninsula de la Guajira. The digital images were transformed to an 8-bit grey scale for computation and were translated to degrees Celsius at the time of presenting the results.

The upwelling system at the Caribbean Colombian coasts is linked to the dynamics of the Atlantic ITCZ. This is defined as a narrow zonal strip where atmospheric convergence of both hemispheres occurs. The generation of the westward trade winds conforms to the Caribbean low-level jet (CLLJ) [8,9,10,11]. The latitude of the ITCZ spans from 4°N to 13°N. From December to April, the ITCZ is in its southernmost position at around 4°N, causing intensification of the trade winds and moving them over the southern Caribbean Sea, generating the main upwelling episode. A full discussion can be found in [3,4,7,8,9,10,11,17].

**Figure 2:**
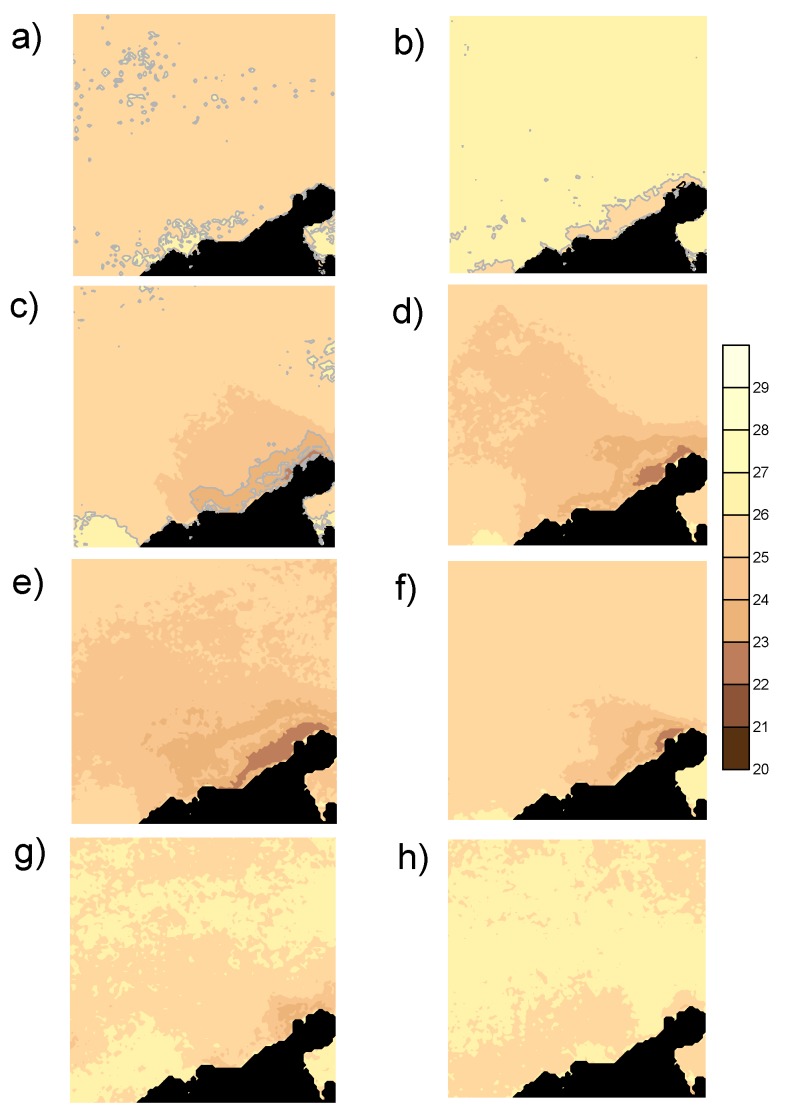
Sequence of sea surface temperature (SST) imagery from (**a**) November 2007, (**b**) December 2007, (**c**) January 2008, (**d**) February 2008, (**e**) March 2008, (**f**) April 2008, (**g**) May 2008, and (**h**) June 2008. Units are degrees Celsius. The coordinates are detailed in Figure 1.

An example of the upwelling event from November 2007 to June 2008 is shown in Figure 2. The image of November 2007 (Figure 2a) is the initial stage with no upwelling conditions (weak westward trade wind) and a high SST close to the coast. The image of December 2007 (Figure 2b) shows how the upwelling starts. The growth of the area with colder waters is very quick in January 2008 (Figure 2c), with a coastal core temperature of about 23 °C. This becomes larger in February (Figure 2d), March (Figure 2e), and April (Figure 2f). Finally, when the ITCZ goes northward, deactivating the episode, the CLLJ takes place and activates the mid-year event (Figure 2g,h).

### 2.2. The Karhunen–Loève Transform

The KLT was independently proposed by [18,19]. The method is widely known under a variety of names, but the main basis is the so-called Hotelling transform [20], also known as principal component analysis [21,22]. The KLT is a commonly used tool to project a time series of a two-dimensional scalar field onto a double empirical-linked basis conformed by self-oscillation spatial modes and their corresponding temporal weights. Decreasing the dimensions of the problem was one of its first applications [23], and it is commonly used for the denoising of time series of two-dimensional fields or for facial and pattern recognition [24,25,26].

In the frame of the KLT, each scene of a time series of equally sampled digital images is named *snapshot*, $\Phi_{n}(\underline{x},t)$, where $\underline{x}$ represents the position vector and *t* stands for time. Their ensemble, $\Phi (\underline{x},t)={\left\{\Phi_{n}(\underline{x},t)\right\}}_{n=1,m}$, is named *super-snapshot,* and it must be decomposed of using the KLT. Let a super-snapshot of *n* be centered on two-dimensional digital images $\Phi (\underline{x},t)-\bar{\Phi }(\underline{x})$. The mean field is $\bar{\Phi }(\underline{x})$. Consider the decomposition of the n-th snapshot, $\Phi_{n}(\underline{x},t)$, in terms of the sum of an orthonormal basis of *m* spatial modes ${\left\{\Psi_{i}(\underline{x})\right\}}_{i=1,m}$, a time-dependent basis ${\left\{A_{i}(t)\right\}}_{i=1,m}$ and a factor to measure the importance (a singular value) of each i-th mode, *M_i_*, in such a way that

(1) $$\Phi_{n}(\underline{x},t)=\sum_{i=1}^{m}M_{i}A_{i}(t)\Psi_{i}(\underline{x})$$

The product of the i-th temporal and spatial terms is the i-th mode. However, it is convenient to compute them separately. The usual dual estimates are

(2) $$A_{i}(t)=\frac{1}{M_{i}}\langle \Psi_{i}\Phi (\underline{x},t)\rangle \Psi_{i}(\underline{x})=\frac{1}{M_{i}}\langle A_{i}(t)\Phi (\underline{x},t)\rangle$$

where <> stands for the projection operator in the Hilbert space [27]. This can be also written as an eigenproblem:

(3) $$\Lambda_{i}A_{i}(t)=\sum_{t'}C_{tt'}A_{i}(t')$$

where the i-th eigenvalue $\Lambda_{i}$ is related to the i-th singular value $M_{i}$ by $\Lambda_{i}=M_{i}^{2}$ and the symmetric, positively defined covariance matrix $C_{tt'}=\langle \Phi (\underline{x},t)\Phi (\underline{x},t')\rangle$. The latter is computed as the autocorrelation function of the super-snapshot and stored in matrix form.

The easiest approach to carry out the eigen-decomposition of Equation (3) is to consider the expression of one snapshot in terms of Equation (1):

(4) $$\phi_{p}(\underline{x},t)=\sum_{i=1}\mu_{i}a_{i}^{}(t)\psi_{i}(\underline{x})$$

where $a_{i}^{}(t)$ and $\psi_{i}(\underline{x})$ are the i-th temporal and spatial modes, respectively, and $\mu_{i}^{}$ is the associated singular value that gives the importance of the i-th mode. The above self-covariance matrix is then written by means of singular value decomposition:

(5) $$C_{tt'}=\int_{0}^{t}dt\int \left(\sum_{k}\mu_{k}a_{k}^{}(t)\psi_{k}(\underline{x})\right)\left(\sum_{l}\mu_{l}a_{l}^{}(t')\psi_{l}(\underline{x})\right)dx=\int_{0}^{1}\left(\sum_{k}\lambda_{k}a_{k}^{}(s)a_{k}^{}(s)\right)ds$$

taking advantage of the ortho-normality of each two bases. The identity described by Fukunaga [22] for the temporal covariance matrix is easily reached:

(6) $$C(s,t)=\sum_{k}\lambda_{k}a_{k}^{}(s)a_{k}^{}(t)$$

where $\lambda_{k}=\mu_{k}^{2}$ is the k-th eigen-value. Finally, the eigen-functions are computed by writing the self-covariance matrix by using a combination of the temporal correlation between times *t* and *t‘* and the spatial modes weighted by their variances:

(7) $$\Psi_{m}(\underline{x})=\frac{1}{\mu_{m}}\sum_{t'}a_{m}(t')\sum_{i=1}\lambda_{i}a_{i}^{}(t)\psi_{i}(\underline{x})$$

The following remarks could be useful for clarifying the above exposition and for computational details. As usual, in the methodology of the principal component analysis, the variables must be centered. The mean field must be computed pixel-by-pixel to prevent bias error through the averaging of undesired areas. So, the first result from the KLT is the mean field, $\bar{\Phi }(\underline{x})$, the second result is the set of spatial modes, and the third is the set of temporal modes. In addition, the spectrum of the eigenproblem sheds light on how many modes must be considered.

The spatial modes give the spatial structure of the oscillation and the intensity (amplitude) of the spatial variation. These modes have time-invariant lines (zero amplitude) or nodal lines, and the field pivots around them. The temporal modes give the corresponding time evolution. They allow the correlation to any exogenous variable to identify external influences if required. The importance of each mode allows us to simplify the problem and filter out the important amount of noise that is always present in any geophysical signal.

Another question is how many eigenmodes must be computed. When a set of *N* images is submitted to a KLT, *N* modes are computed, as in the principal component analysis. However, this may be not the best option, because the first eigenvalue is sensitive to round-off errors, and its correction is very difficult. It is convenient to think that the covariance matrix holds the signal and noise subspaces and that the eigenmodes to be computed are assigned to the signal subspace. From a practical point of view, the number of eigenmodes can be one-fifth, and perhaps one-quarter, of the number of available images.

### 2.3. Time-Series Prediction Models

#### 2.3.1. Parametric Time-Series Models

After Box–Jenkins, the most celebrated parametric time-series model is the auto-regressive integrated moving averaged (ARIMA) model [28,29]. The integration is introduced to take trends into account, and the AR and MA terms are devoted to the so-called deterministic and noise parts, respectively. The temporal modes from the KLT oscillate around a mean value with no trend, so the integration is not needed, and it leads to the ARMA(p,q) model:

(8) $$x\left(t\right)=1+\overset{p}{\underset{n=1}{\sum }}a_{n}\cdot x_{t-n}+\overset{q}{\underset{n=1}{\sum }}b_{n}\cdot \theta_{t-n}$$

where *p* and *q* represent the order of the AR and MA parts, respectively; *a_n_* represents the reflection coefficients; *b_n_* represents the coefficients of the MA part; and *θ_t_* is the noise at time *t*. The coefficients are computed by the Yule–Walker equations, and they must verify the Levinson recursion to ensure that the roots of the polynomials in Equation (8) lie in the unit circle [28,29].

The parametric time-series models are barely constrained by the parsimony principle. This means that the order of the model must be the lowest possible [28,29]. Once the coefficients of Equation (8) are known, the prediction is easily computed.

#### 2.3.2. Harmonic Prediction Model

The harmonic prediction model in based on the frequency composition of the time series. Because the time series of the modes are short, the Fourier transform is not efficient for computing the power spectral density due to the spectral leakage and low resolution [15]. The maximum entropy spectral analysis (MESA) was used instead. This consists of fitting the autoregressive parametric model

(9) $$x\left(t\right)=1+\overset{P}{\underset{n=1}{\sum }}a_{n}\cdot x_{t-n}.$$

By taking its z-transform and making a sine–cosine recursion, the result is a Fourier-like decomposition with very sharp peaks and very well located frequencies [15]. Once the spectral information has been computed and the frequency composition is known, the harmonic least-squares fit is

(10) $$w\left(t\right)=w_{0}+\overset{p}{\underset{i=1}{\sum }}\left[a_{i}\cdot \cos\left(\omega_{i}t\right)+b_{i}\cdot \sin\left(\omega_{i}t\right)\right]$$

which gives the mean value *w_0_* and the amplitudes of the circular functions *[a,b]_i_*. Now, it is possible to compute a prediction at a given time.

The key point is the determination of the order of the AR model. This is not constrained to the parsimony principle, and the order of the AR model can be chosen to be as high as needed from the considerations of Wold’s Theorem [28]. The upper limit occurs when spontaneous line splitting is observed [15].

## 3. Results and Discussion

### 3.1. KLT

**Figure 3:**
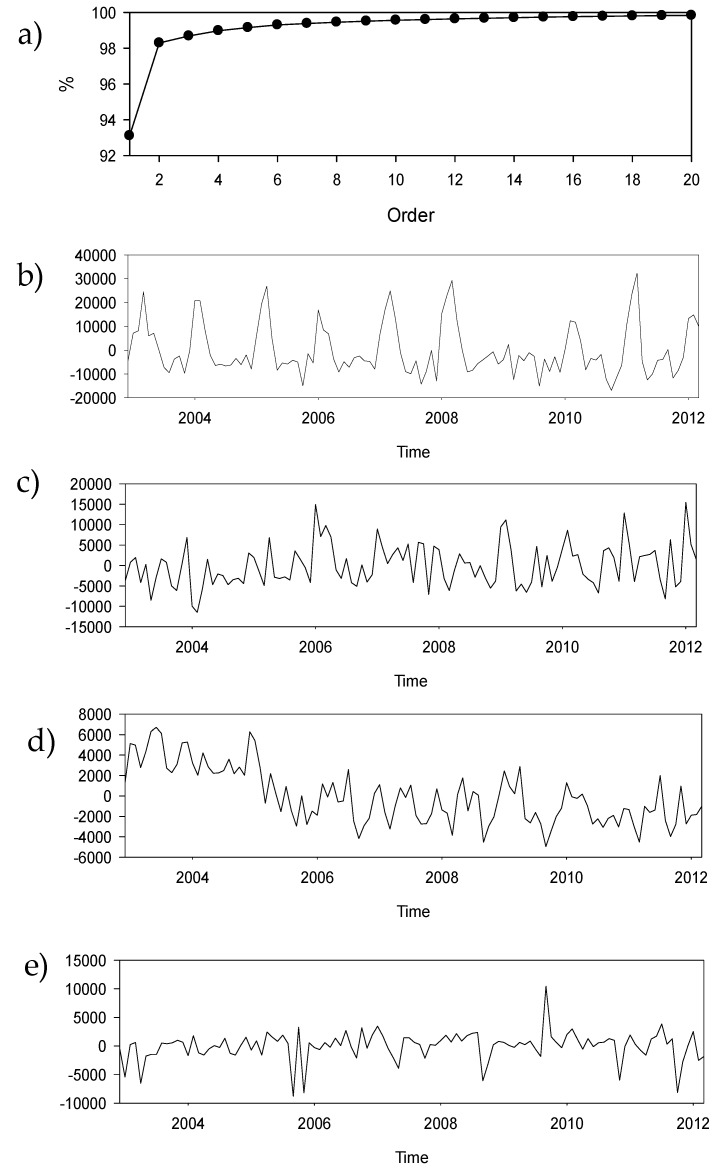
(**a**) Cumulative percentage of explained variance; (**b**–**e**) first four temporal modes from the Karhunen–Loève transform (KLT) decomposition of SST imagery.

The first 112 available MODIS-SST images (December 2002 to March 2012) were processed using the KLT (Section 2.2). The cumulative explained variance was computed from the spectrum of the eigenproblem (Figure 3a). The first mode explained up to 93% of the whole variance, and the first five modes explained close to 99%. The temporal weights of the first four are presented in Figure 3b–d. The first and most important mode presents a very regular pattern with a period of about one year (Figure 3b). The second and third modes (Figure 3d) have many short-period components and a smooth evolution. The fourth and higher modes are minor modes. It is desirable for the second to fourth modes to increase their explained variance by about 5% and to take into account short-term oscillations for predictions.

**Figure 4:**
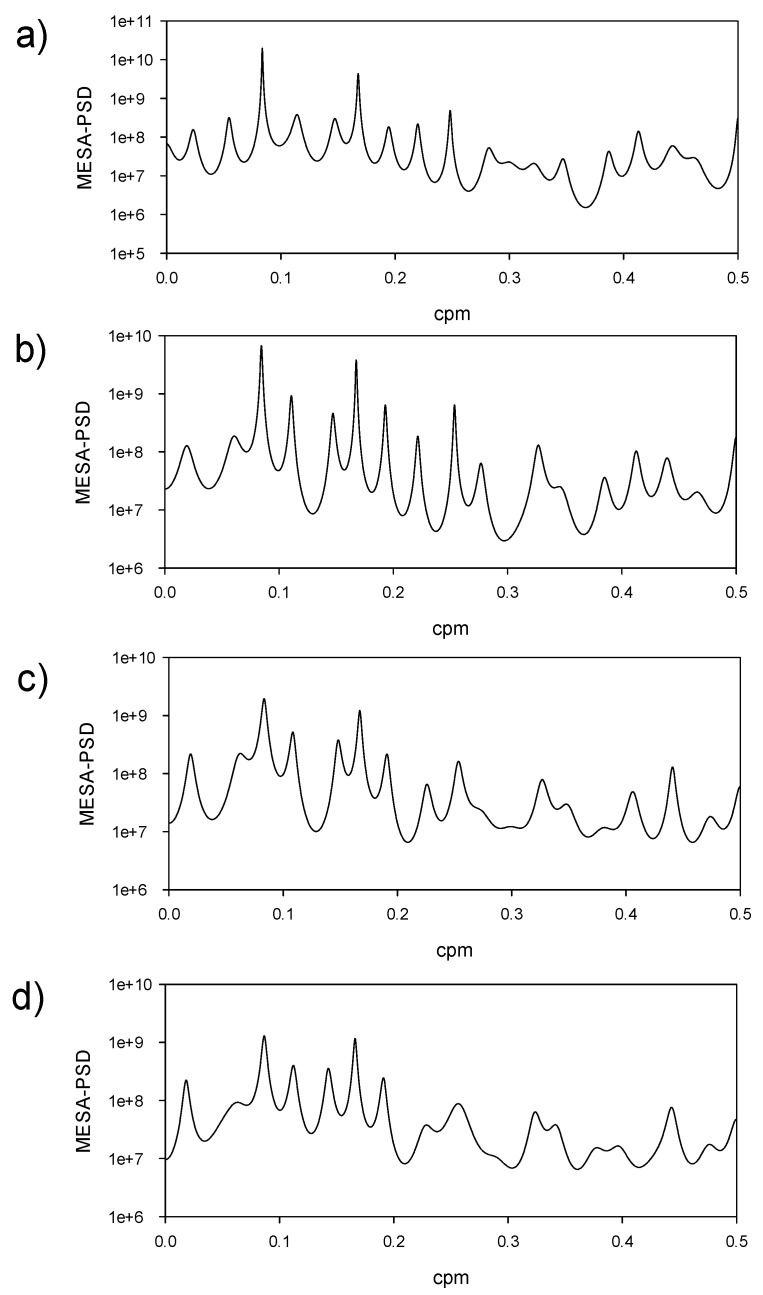
Logarithmic power spectral density computed by maximum entropy spectral analysis (MESA-PSD) of the first four temporal modes. The frequency is cycles per month (cpm).

The logarithmic MESA-PSD values of the first four temporal modes are shown in Figure 4a–d, and more relevant periods can be found in Table 1. The first and most important mode (Figure 4a) presents a well-defined temporal pattern (Figure 3b) with a period of one year and six months (Figure 4a). The second mode (Figure 4b) presents the same periods and some others, such as 9, 6.8, 5.2, and 4 months. The third and fourth modes (Figure 4c,4d) have the same periods in their compositions, and a new one of 54 months long is detected. The only clear periods for reading are the annual and semiannual periods; they are used in most of the geophysical signals. The other periods are intentionally left for further works, but some of them were previously noted in [8] and [10].

**Figure 5:**
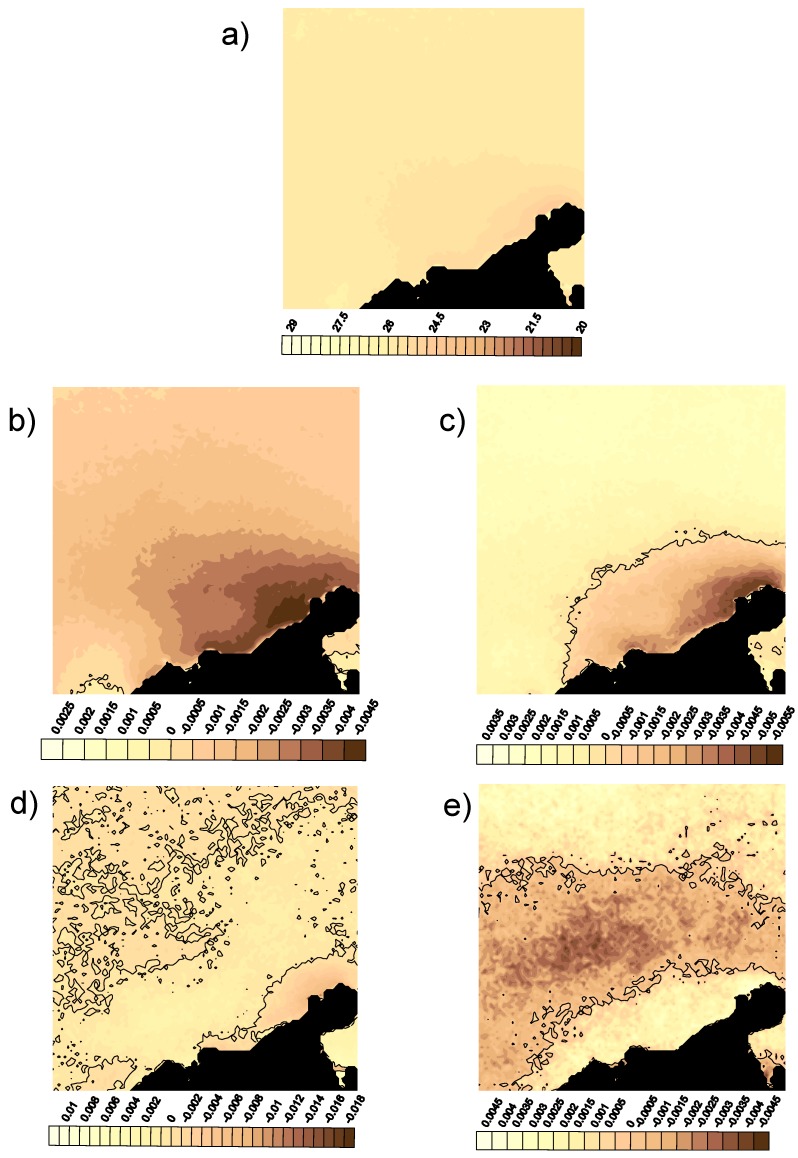
Mean SST field and the first five spatial modes from the KLT decomposition: (**a**) mean SST field in degrees Celsius; (**b**–**e**) represent spatial modes 1 to 4, respectively. The continuous lines in the spatial modes are the zero-value level, or the nodal lines.

The mean SST field and the first four spatial modes are presented in Figure 5. Because the variables submitted to a KLT must be centered, one of the results of the KLT is the average SST field (Figure 5a). It shows a permanent structure at Punta Gallinas and along the Colombian coast, but not at Cabo de la Aguja. The mean value at the upwelling cores is about 22–23 °C, and when there is no upwelling, it is about 28–29 °C. The first mode (Figure 5b) presents the structure of the developed upwelling. The maximum values follow the coast, and all values have the same sign, except at the mouth of Lake Maracaibo and around Santa Marta. The second mode (Figure 5c) presents two differentiated areas. The first is close to the coast, and the other takes the open sea into account, both with different signs in their values. The third and fourth modes (Figure 5d,e) show small scattered structures that can be thought of as noise. Although the fourth mode (Figure 5d) reveals a nice spatial structure, it is necessary to keep in mind that it has a small contribution to the explained variance (Figure 3a).

The zero value lines, the nodal lines, are also represented in the spatial modes (Figure 5). The time-invariant lines in a given mode are where each spatial mode pivots, and they can be considered as the statistical limits of the upwelling events in a given area. The most important mode, i.e., the first one in Figure 5b, has no nodal lines, and all fields will oscillate up and down with the corresponding temporal mode. The highest amplitudes cover the area affected by the upwelling. The second mode (Figure 5c) has a nodal line dividing the domain into two halves with different signs, which oscillate up and down depending on the temporal weight. The highest amplitudes correspond to the areas with the coldest waters, and the nodal line can be considered the limit of the most important part of the upwelling. The third mode (Figure 5d) presents some noise, and the fourth mode (Figure 5e) has a banded structure with three areas that are well-delimited by two nodal lines.

### 3.2. A Prediction Model for the Upwelling Events

The advantage of the KLT is the drastic reduction in complexity, because the modes are considered separable in time and space (see Section 2.2). So, it is possible to build up a simple prediction model from the temporal modes without getting into the complexities of a numerical model. Although the predicting method is customary, its effectiveness depends on the experience of the researcher in time-series analysis. The set of 119 digital images was divided in two. The first 112 images (December 2002 to March 2012) was the training set, and it was submitted to a KLT decomposition. The temporal modes were predicted by two different methods, and their compositions (Equation (4)) were compared to the last six images (April 2012 to September 2012).

The worst situation in which to test the methodology is when the upwelling is deactivated and the warmer water of the open Caribbean reaches the Colombian coasts. Two methods were used to test this unfavorable situation. The first one consists of prediction using the autoregressive-moving average (ARMA) parametric time-series model. An ARMA(2,1) (see Section 2.3) was estimated for most of the temporal modes after the analysis of the autocorrelation and partial autocorrelation functions [28,29] and six projected time steps. One of the temporal modes was fitted to an AR(2) because the MA part could not be computed. The predicted SST field was computed from Equation (4) considering the prediction of the temporal factor and adding the mean SST field. The second method used for prediction was the harmonic method. The spectral information of the temporal modes was computed using MESA (Figure 4 and Table 1). Then, the least-squares method in Equation (8) led to the prediction model.

**Figure 6:**
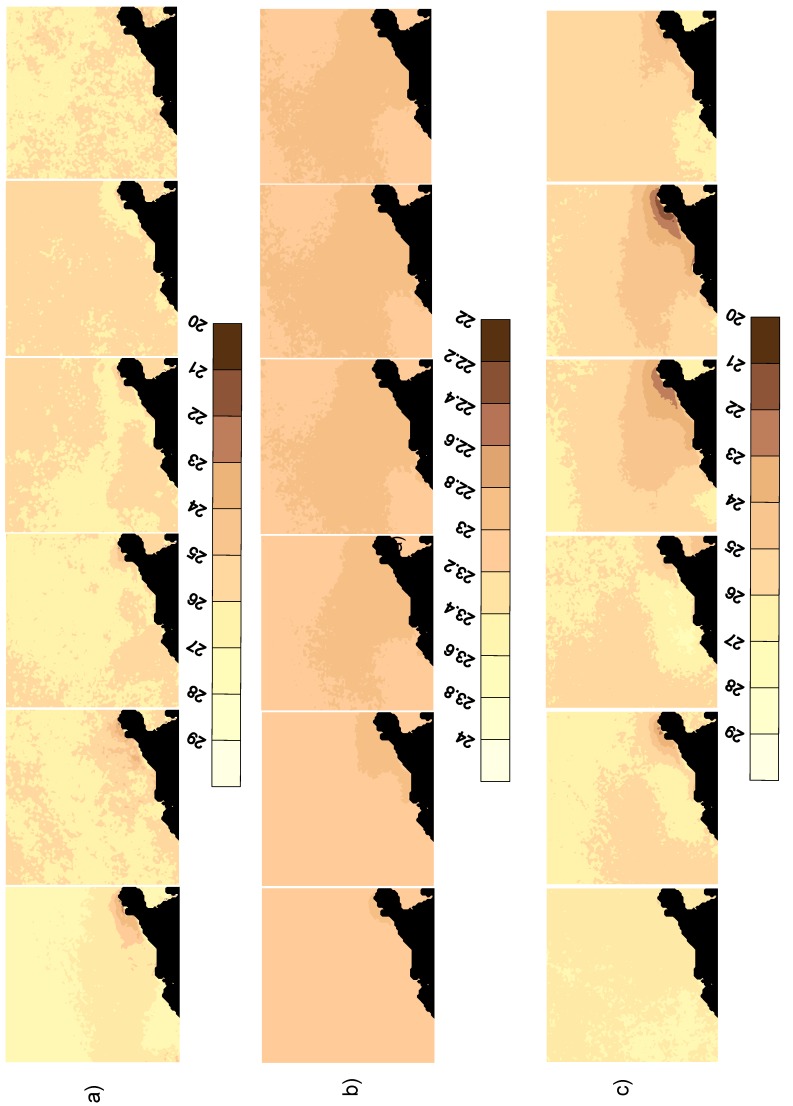
(**a**) The observed SST scenes from April 2012 to September 2012; (**b**) predicted SST field from autoregressive-moving average (ARMA) models for the same months; (**c**) predicted SST field from harmonic synthesis for the same months. Units are degrees Celsius.

Figure 6 presents the MODIS-SST images for April to September 2012 (Figure 6a, from left to right), and the predictions of the same scenes using the ARMA model are presented in Figure 6b. The same parameters with harmonic synthesis are presented in Figure 6c. The first inspection reveals that the parametric time-series ARMA mode failed in the prediction, because the temperature range was quite narrow and far from the observed imagery. The harmonic method performed better, and its solutions were smoother, with results close to the observed data.

The correlations between the original MODIS-SST images and the predictions are presented in Table 2. The land was kept out of the analysis to avoid biased results. The goodness of fit was extremely good for the ARMA models (>0.9) for the months of April and May, and the harmonic prediction the worst (about 0.8). This is because there was no upwelling and no organized structures. However, for the rest of the months, when a weak upwelling episode started, the ARMA model predictions were the worst, falling under 0.75, lower than the harmonic prediction (which was consistently about 0.8). It may be concluded that the harmonic method works better for longer time predicting horizons and when there are organized structures.

## 4. Conclusions

The Colombian Caribbean upwelling system is almost permanent due to the combination of the morphology of the coast, the bathymetry, and the persistently blowing westward trade winds related to the dynamics of the ITCZ. The KLT is very useful for simplifying the analysis of a time series of digital images to obtain three types of information: the spectrum of the problem, the temporal modes, and the spatial modes. The spatial modes give information on the intensity of the spatial variations and the very important nodal lines. These show a banded spatial structure with an area near the coast, another at the open sea, and the third as a transitional area in the middle. The visual inspection of the temporal weights revealed periodic patterns in many modes.

Two time-series prediction models were tested. The ARMA model presented a very high correlation (>0.9) when there were no upwelling events; otherwise, it must be not considered. The harmonic synthesis performed much better under upwelling conditions (>0.8). The horizon of prediction depended on how developed the upwelling event was, but the harmonic prediction worked better than the parametric ARMA model. The prediction models could be improved by considering additional variables in the models with the contribution of exogenous variables as climate indexes (see [8,10] for two examples).

## Figures and Tables

**Table 1:** Relevant components for the first four SST modes.

Mode	Period (Months)
1	11.9, 6.8
2	11.9, 9.0, 6.8, 6.0, 5.2, 4.0
3	54.3, 11.9, 9.0, 6.8, 6.0, 5.2, 4.0
4	54.3, 11.9, 9.0, 6.8, 6.0, 5.2, 4.0

**Table 2:** Correlations of the predictions from autoregressive-moving average (ARMA) and harmonic models with the MODIS-SST images for the six last months of the imagery set (April to September 2012).

Months	ARMA	Harmonic
April	0.9760	0.8130
May	0.9103	0.8154
June	0.7278	0.8091
July	0.5855	0.8007
August	0.4366	0.7939
September	0.5123	0.8043
